# Adapting Recruitment and Retention for Korean American Older Adults in a Community Clinical Trial During COVID-19

**DOI:** 10.1093/geroni/igaf122.364

**Published:** 2025-12-31

**Authors:** Ji-Young Yun, Deborah Min, Ji-Young Cho, Sara Kim, Hae-Ra Han

**Affiliations:** Johns Hopkins University, Wellesley, Massachusetts, United States; Johns Hopkins Bloomberg School of Public Health, United States; Korean Community Service Center of Greater Washington, Annandale, Virginia, United States; Korean Community Services of Metropolitan New York, Bayside, New York, United States; Johns Hopkins University, Baltimore, Maryland, United States

## Abstract

Recruiting and retaining underrepresented populations, particularly those with limited English proficiency, remains challenging in clinical trials, especially during public health crises like COVID-19. The PLAN study (Preparing Healthy Aging Through Dementia Literacy Education and Navigation), a community-based clinical trial, contacted 2,997 Korean American older adults for cognitive screening between February 2021 and January 2024, enrolling 288 dyads of Korean American older adults with probable dementia and their caregiver (N = 576). The study adapted recruitment and retention strategies to evolving pandemic conditions. In the first phase (Feb–Jul 2021), strict COVID-19 protocols limited outreach to virtual methods, engaging 461 dyads. The second phase (Aug 2021–Mar 2023) combined virtual and in-person approaches, reaching 1,981 dyads as restrictions eased. The final phase (Apr 2023–Jan 2024), post-pandemic, engaged 555 dyads primarily through in-person efforts. Recruitment strategies included social media, ethnic media, and outreach at adult daycare centers, churches, and senior centers. Virtual methods, such as ethnic newspapers and KakaoTalk, were effective early on, while in-person engagement—particularly through adult daycare centers and word-of-mouth referrals—proved more successful later. Virtual recruitment yielded a 5% success rate, compared to 10% for in-person efforts. Retention strategies, including monetary compensation and consistent communication, contributed to a low 6% dropout rate. This study underscores the importance of population-tailored recruitment strategies and provides valuable insights into optimizing outreach for future community-based clinical trials.

